# Emerging Role for Acyl-CoA Binding Domain Containing 3 at Membrane Contact Sites During Viral Infection

**DOI:** 10.3389/fmicb.2020.00608

**Published:** 2020-04-08

**Authors:** Yue Lu, Siqi Song, Leiliang Zhang

**Affiliations:** ^1^School of Medicine and Life Sciences, University of Jinan-Shandong Academy of Medical Sciences, Jinan, China; ^2^Institute of Basic Medicine, The First Affiliated Hospital of Shandong First Medical University, Jinan, China; ^3^School of Basic Medicine, Qingdao University, Qingdao, China

**Keywords:** ACBD3, PI4KB, membrane contact sites, replication organelles, picornavirus

## Abstract

Acyl-coenzyme A binding domain containing 3 (ACBD3) is a multifunctional protein residing in the Golgi apparatus and is involved in several signaling pathways. The current knowledge on ACBD3 has been extended to virology. ACBD3 has recently emerged as a key factor subverted by viruses, including kobuvirus, enterovirus, and hepatitis C virus. The ACBD3-PI4KB complex is critical for the role of ACBD3 in viral replication. In most cases, ACBD3 plays a positive role in viral infection. ACBD3 associates with viral 3A proteins from a variety of *Picornaviridae* family members at membrane contact sites (MCSs), which are used by diverse viruses to ensure lipid transfer to replication organelles (ROs). In this review, we discuss the mechanisms underlying the involvement of ACBD3 in viral infection at MCSs. Our review will highlight the current research and reveal potential avenues for future research.

## Introduction

Ten years ago, PI4KB (for enterovirus) and PI4KA (for hepatitis C virus, HCV) were identified as host factors to produce phosphatidylinositol-4-phosphate (PI4P) for virus replication (Hsu et al., 2010; Reiss et al., 2011). Subsequently, PI4KB was discovered as the target of a group of potent antiviral candidates (major enviroxime-like compounds) with unknown target for about 30 years (Arita et al., 2011; Delang et al., 2012), which confirmed the importance of PI4KB in enterovirus replication and in antiviral development.

Acyl-coenzyme A binding domain containing 3 (ACBD3), is a multifunctional protein that resides in the Golgi apparatus and is mainly involved in the maintenance of the Golgi apparatus structure and the regulation of intracellular transport between the endoplasmic reticulum (ER) and the Golgi apparatus (Sohda et al., 2001). ACBD3 also regulates the synthesis of fatty acyl-coenzyme A (Chen et al., 2012). ACBD3 contains several functional domains, including an acyl-coenzyme A binding (ACB) domain, a coiled-coiled domain, a glutamine-rich domain (Q-domain), and a Golgi dynamic domain (GOLD domain) (Klima et al., 2016). Three groups independently identified ACBD3 as a binding partner of viral 3A proteins by mammalian two-hybrid screening (Sasaki et al., 2012), yeast two-hybrid screening (Teoule et al., 2013), or affinity purification coupled with mass spectrometry (AP-MS) (Greninger et al., 2012). Identification of ACBD3 attracted intense attention for its importance as a potential hub between PI4KB and viral proteins in viral replication. However, the following studies did not necessarily consistent with this original concept of the role of ACBD3, and currently the role of ACBD3 is rather ambiguous.

The concept of membrane contact sites (MCSs) in virus infection was established by the discovery of oxysterol-binding protein (OSBP) as the target of minor enviroxime-like compounds (Arita et al., 2013), and as the effector of PI4KB (Arita, 2014) or of PI4KA (Wang et al., 2014) in virus replication. MCSs are regions where the membranes of two organelles come into close proximity to facilitate communication of the organelles with each other. To integrate compartmentalized cellular functions, MCSs promote non-vesicular exchange of lipids and ions. Many viruses remodel host membranes into specialized membranous replication organelles (ROs) to facilitate viral replication. Virus-induced MCSs (vMCSs) generate ROs by supporting the synthesis and redistribution of lipids, which requires a number of proteins at MCSs, such as OSBP, PI4KB, and ACBD3.

It has been found that ACBD3 recruits PI4KB to the Golgi and trans-Golgi network (TGN) membranes and increases PI4KB enzymatic activity to produce PI4P locally (Baumlova et al., 2014; Boura and Nencka, 2015). PI4P regulates the docking of OSBP to the Golgi, which in turn delivers cholesterol from the ER to the Golgi. In the last decade, researchers have discovered that PI4P is necessary for viral replication in many viruses (Graham and Burd, 2011). Those viruses hijack the ACBD3 protein to recruit PI4KB to the viral ROs to produce PI4P (Hsu et al., 2010; Ronnberg et al., 2012; Nchoutmboube et al., 2013). The aim of this mini-review is to summarize the role of ACBD3 at MCSs during viral infection. By describing the interaction between viral proteins and ACBD3, we will reveal the diversity of the interplay between ACBD3 and viruses, offering a broad perspective on this emerging host–virus interaction.

## ACBD3 in Kobuvirus Infection

Aichi virus (AiV), a member of the kobuvirus genus in the *Picornaviridae* family, is one of the pathogenic factors of gastroenteritis. Researchers demonstrated that the non-structural proteins 2B, 2BC, 2C, 3A, and 3AB from Aichi virus interacted with ACBD3 and PI4KB to form a protein complex at ROs to promote PI4P synthesis (Greninger et al., 2012; Sasaki et al., 2012; Klima et al., 2017; McPhail et al., 2017; Chalupska et al., 2019). Silencing of ACBD3 or PI4KB inhibited viral replication by about 70% (ACBD3) or 99% (PI4KB). Expression of the viral proteins 2B, 2BC, 2C, 3A, and 3AB alone could promote PI4P synthesis (Ishikawa-Sasaki et al., 2014). In cells that are not infected with AiV, the C-terminal sequence of ACBD3 binds to the cytoplasmic region of the giantin C-terminal, and giantin is anchored to the Golgi membrane through the C-terminal anchor domain. PI4KB localizes to the Golgi apparatus by interacting with ACBD3. In cells infected by AiV, the viral proteins 2B, 2BC, 2C, 3A, and 3AB compete with Golgi giantin to bind to ACBD3, causing viral protein/ACBD3/PI4KB formation, and the colocalization of giantin and ACBD3 disappears (Greninger et al., 2012; Sasaki et al., 2012; Klima et al., 2017; McPhail et al., 2017; Chalupska et al., 2019).

Researchers have shown that the AiV non-structural protein 3A plays an important role in membrane rearrangement and inhibition of the host cell ER-to-Golgi transport pathway (Greninger et al., 2012). When it binds to the GOLD domain of ACBD3, the intrinsically disordered protein AiV 3A adopts a highly ordered structure and is targeted to the membrane (Klima et al., 2017; McPhail et al., 2017). Then, 3A recruits and activates PI4KB, resulting in the production of PI4P (Chalupska et al., 2019). Researchers have analyzed the conformation of the ACBD3 protein and viral 3A protein in solution by using small angle X-ray scattering (SAXS) and computer simulation (Rozycki and Boura, 2014; Peti et al., 2018). Both the ACBD3 protein and the 3A:ACBD3 protein complex exhibit extended and flexible conformations in solution (Chalupska et al., 2019).

Interestingly, cholesterol accumulates on the AiV ROs via protein-protein interactions of VAP/OSBP/SAC1 with the AiV proteins and with ACBD3 (Ishikawa-Sasaki et al., 2018). OSBP, VAP-A/B, SAC1, and PITPNB are well-known components of the cholesterol transport pathway. Silencing of these proteins reduced AiV replication, indicating the involvement of the cholesterol transport pathway in AiV RNA replication (Ishikawa-Sasaki et al., 2018). Based on the interactions between ACBD3 and the component proteins of the cholesterol transport pathway, ACBD3 is defined as a novel component of this pathway.

## ACBD3 in Enterovirus Infection

### Enterovirus A71 (EV-A71)

Enterovirus A71 (EV-A71), a member of the EV-A species of the *Picornaviridae* family, is one of the causative agents of hand, foot, and mouth disease (HFMD) and induces neurological complications such as aseptic meningitis and brainstem and cerebellar encephalitis (Solomon et al., 2010; Xing et al., 2014). EV-A71-induced PI4P production is dependent on PI4KB and ACBD3 (Xiao et al., 2017). EV-A71 3A associates with the GOLD domain of ACBD3. Silencing of ACBD3 by siRNA or knockout of ACBD3 inhibited the replication of EV-A71 by 70% (siRNA) or nearly 100% (knockout) in RD cells, suggesting that ACBD3 is critical for EV-A71 replication (Lei et al., 2017). Silencing of PI4KB by siRNA or knockout of PI4KB suppressed the replication of EV-A71 by about 80% or by about 95%, indicating the key role for PI4KB in EV-A71 replication (Xiao et al., 2017). EV-A71 3A promotes the formation of a stable ACBD3-PI4KB complex (Lei et al., 2017). I44A or H54Y substitution in EV-A71 3A interrupted the interaction between 3A and ACBD3 (Lei et al., 2017). Moreover, I44 and H54 are important for stabilizing the ACBD3-PI4KB complex and are critical for EV-A71 replication (Xiao et al., 2017). Surprisingly, a recent study on trans-rescue of EV-A71 pseudovirus replication with PI4KB deletion mutants suggested that ACBD3-binding site of PI4KB is not essential for EV-A71 replication (Arita, 2019). The role of ACBD3-PI4KB interaction involved in EV-A71 infection need to be further investigated.

### Coxsackievirus (CV)

Coxsackievirus (CV), a member of the EV-B species of the *Picornaviridae* family, infects the human body through the respiratory tract and digestive tract. After infection, people exhibit cold symptoms such as fever, sneezing and coughing. Infection during pregnancy can cause non-paralytic poliomyelitis, intrauterine infection and teratogenicity of the fetus. CV is divided into groups A and B. Early studies have suggested that the recruitment of PI4KB to the CVB3 RO has nothing to do with GBF1/ARF1 and ACBD3 (White and Aitken, 1989; van der Schaar et al., 2012; Dorobantu et al., 2014). The GOLD domain of ACBD3 directly interacts with CVB3 3A (Greninger et al., 2012; Dorobantu et al., 2014). Silencing of ACBD3 did not affect the recruitment of PI4KB to the RO by 3A (van der Schaar et al., 2012; Klima et al., 2017). Recent studies showed that CVB3 enhanced the recruitment of PI4KB through binding to GBF1/ARF1 and interaction with ACBD3 (Klima et al., 2017). A previous study did not observe an effect on ACBD3 replication caused by CVB3 in HeLa cells with more than 90% knockdown (Dorobantu et al., 2014, 2015). However, 100% knockdown by CRISPR reduced CVB3 replication by more than 90% in HeLa cells (Lyoo et al., 2019). Overall, the current opinion is that ACBD3 promotes CVB3 replication.

### Poliovirus (PV)

Poliovirus (PV), a member of the EV-C species of the *Picornaviridae* family, invades the central nervous system, damages motor nerve cells in the anterior horn of the spinal cord, and causes limb relaxation paralysis, which is often observed in children. PV proteins modulate PI4KB activity and thus provide PI4P for recruitment of OSBP to accumulate with unesterified cholesterol in ROs (Arita, 2014). The PV protein 3A interacts with ACBD3, and silencing of ACBD3 reduced PV replication in HeLa cells (Greninger et al., 2012). Subsequent experiments showed that ACBD3 inhibited PVS2 PV and PVS2 recombinant virus expressing the CV-A17 virus 3A in HEK-293T, IMR5, and HeLa cells (Teoule et al., 2013). Recently, researchers investigated the role of ACBD3 in PV replication by the CRISPR-Cas9 technique in HeLa cells (Lyoo et al., 2019). They demonstrated that ACBD3 promoted PV replication by more than 10-fold (Lyoo et al., 2019). A recent study on trans-rescue of PV1 pseudovirus replication with PI4KB deletion mutants showed that ACBD3-binding site of PI4KB is not essential for PV1 pseudovirus replication (Arita, 2019). Whether the interaction of ACBD3-PI4KB is involved in *bona fide* PV1 infection need more studies to clarify.

### Enterovirus D68 (EV-D68)

Enterovirus D68 (EV-D68), a member of the EV-D species of the *Picornaviridae* family, is an emerging respiratory pathogen. EV-D68 3A associated with ACBD3 and enhanced the ACBD3-PI4KB interaction. Silencing of ACBD3 by siRNA or knockout of ACBD3 suppressed EV-D68 replication by about 60% (siRNA) or about 90% (knockout) in RD cells, suggesting that ACBD3 is critical for EV-D68 replication (Lei et al., 2017). Knockout of PI4KB suppressed the replication of EV-D68 by about 95%, indicating the key role for PI4KB in EV-D68 replication (Xiao et al., 2017).

The crystal structure of the complex of the ACBD3 GOLD domain and EV-D68 3A indicated that the GOLD-3A interaction was mediated through multiple hydrophobic interactions and hydrogen bonds (Horova et al., 2019). The alpha helices P19-V29 in 3A (α1^3A^) and Q32-K41 in 3A (α2^3A^) interacted with a shallow cavity of the GOLD domain formed by antiparallel beta strands of ACBD3. The beta strand V53-I58 in 3A (β2^3A^) associated with the strand V402-P408 in ACBD3, while the beta strand I44-I46 in 3A (β1^3A^) interacted with the strand K518-R528 in ACBD3 (Horova et al., 2019). Because no direct interaction between PI4KB and the enterovirus 3A proteins has been identified, the ACBD3-PI4KB interaction stimulated by 3A and the subsequent enhancement of the membrane-targeting of PI4KB in infected cells depend on the ACBD3-3A association.

### Rhinovirus (RV)

Rhinoviruses (RVs), belonging to the enterovirus genus of the *Picornaviridae* family, are the causative agents for common cold. Over 150 types of RVs are classified into three species: RV-A, RV-B, and RV-C. Studies have shown that the 3A proteins from rhinovirus A2 (RVA2), rhinovirus A16 (RVA16), and rhinovirus B14 (RVB14) (Greninger et al., 2012) interact with ACBD3. Previous studies suggested that silencing of ACBD3 had no effect on RVA2 and RVB14 replication (Dorobantu et al., 2014, 2015). Recently, this conclusion was corrected by a more careful study. ACBD3 knockout inhibits replication of RVA2 and RVB14 by more than 90% in HeLa and HAP1 cells (Lyoo et al., 2019). ACBD3 is important for proper localization of 3A of RVA2 and RVB14. Interestingly, silencing of ACBD3 by siRNA or knockout of ACBD3 increased the replication of HRV16 by about 50% in HeLa cells, suggesting that ACBD3 inhibited RVA16 replication (Xiao et al., 2017).

## ACBD3 in Infections Caused by Other Viruses

### HCV

HCV belongs to the *Flaviridae* family and causes chronic liver diseases, liver cirrhosis and even liver cancer. Studies have shown that PI4P and PI4KA play an important role in HCV infection. HCV NS5A (Tai et al., 2009; Reiss et al., 2011) protein can hijack ARFGAP1 to maintain the PI4P concentration required for replication (Li et al., 2014). However, the role of PI4KB in HCV replication is genotype (GT)-dependent.

ACBD3 knockdown in the OR6 HCV replicon system increased HCV replication by around 70%, while ACBD3 overexpression reduced HCV replication by about 40%, indicating that ACBD3 could inhibit HCV replication (Hong et al., 2014). Further study revealed an interaction between NS5A and ACBD3. It was found that there was a GT-dependent association between NS5A and ACBD3. The binding ability of 1b NS5A to ACBD3 was stronger than that of other subtypes. NS5A associates with the same region of ACBD3 as PI4KB, that is, the amino acid sequence 116–327 of ACBD3. Therefore, NS5A and PI4KB competitively interacted with ACBD3. NS5A could hijack ACBD3 from the PI4KB/ACBD3 complex to form the NS5A/ACBD3 complex and release PI4KB to produce PI4P, which is beneficial for HCV infection (Hong et al., 2014).

### African Swine Fever Virus (ASFV)

African swine fever virus (ASFV) is an acute, febrile and highly contagious virus that causes hemorrhagic fever in wild and domestic pigs with high mortality. It is an enveloped double-stranded DNA virus in the *Asfarviridae* family (Alonso et al., 2018). The length of the ASFV genome is 170–190 kb, which encodes a 151–167 open reading frame (ORF) (Chapman et al., 2008; de Villiers et al., 2010).

ASFV generates viral ROs to amplify its genome, and lipid exchange is the basis for the formation of ROs. ASFV replication requires cholesterol transport mediated by OSBP. Itraconazole (ITZ) targets OSBP and OSBP-related protein 4 (ORP4) to reduce sterol synthesis. 25-Hydroxycholesterol (25-HC) inhibits cholesterol transport by binding OSBP. ITZ at 100 μM reached 65% inhibition of ASFV replication, while 25-HC at 50 μM reduced ASFV replication by about 70% (Galindo et al., 2019). Upon ASFV infection, a number of proteins at MCSs, such as OSBP, PI4KB, and ACBD3, are recruited to ROs, confirming that cholesterol shuttling is required for ASFV RO formation (Galindo et al., 2019). However, whether ACBD3 plays an important role for ASFV replication remains to be investigated.

## Discussion

With the accumulating investigations of the role of ACBD3 in viruses, the mechanisms by which ACBD3 is involved in viral infection are gradually being elucidated. ACBD3 interacts with many 3A proteins from *Picornaviridae* family to affect viral replication (Table 1). Studies have confirmed that ACBD3 has the following effects on viral replication: (1) to promote the infection caused by viruses such as AiV; (2) to inhibit the infection caused by viruses such as HCV.

**TABLE 1:** Summary of the role of ACBD3 in viral replication.

Virus	Family (Genus)	Effect on viral replication	Proposed mechanism	References
Aiv	*Picornaviridae (kobuvirus)*	Promotion	AiV virus protein binds to ACBD3 protein and recruits PI4KB to form virus protein/ACBD3/PI4KB complex to synthesize PI4P for replication.	Sasaki et al., 2012; McPhail et al., 2017
EV-A71	*Picornaviridae (enterovirus)*	Promotion	EV-A71 3A associates with ACBD3. EV-A71 3A promotes the formation of a stable ACBD3-PI4KB complex. ACBD3 is critical for EV-A71 replication.	Xiao et al., 2017
CV	*Picornaviridae (enterovirus)*	Promotion	CVB3 3A interacts with ACBD3. ACBD3 promotes CVB3 replication. CVB3 enhanced the recruitment of PI4KB through interaction with ACBD3.	Lyoo et al., 2019
PV	*Picornaviridae (enterovirus)*	Promotion	Poliovirus protein 3A interacts with ACBD3 and ACBD3 knockout reduces poliovirus replication.	Lyoo et al., 2019
EV-D68	*Picornaviridae (enterovirus)*	Promotion	EV-D68 3A associated with ACBD3 and increased ACBD3-PI4KB interaction. ACBD3 is critical for EV-D68 replication.	Xiao et al., 2017
RVA2, RVB14	*Picornaviridae (enterovirus)*	Promotion	3A proteins from RVA2 and RVB14 interact with ACBD3. ACBD3 is critical for the replication of RVA2 and RVB14.	Lyoo et al., 2019
RVA16	*Picornaviridae (enterovirus)*	Inhibition	3A proteins from RVA16 interact with ACBD3. ACBD3 inhibited RVA16 replication.	Xiao et al., 2017
HCV	*Flaviridae (hepacivirus)*	Inhibition	ACBD3 inhibits HCV replication. NS5A from different GTs of HCV compete with PI4KB to bind ACBD3.	Hong et al., 2014

The results of different studies on the role of ACBD3 in the replication of CV, RV, and PV are not completely consistent. ACBD3 has been recognized as an important but not always essential protein for the replication of enteroviruses. One key reason is the different interference approaches used for knockout and knockdown. In previous studies, researchers applied siRNA technology. It is not possible to completely knock out ACBD3; thus, the remaining small amount of ACBD3 could also support viral replication. With the technical improvements that came with CRISPR method, it is possible to assess the role of ACBD3 in viral replication in the knockout setting. It has become obvious that ACBD3 is an essential factor for these enteroviruses.

**FIGURE 1:**
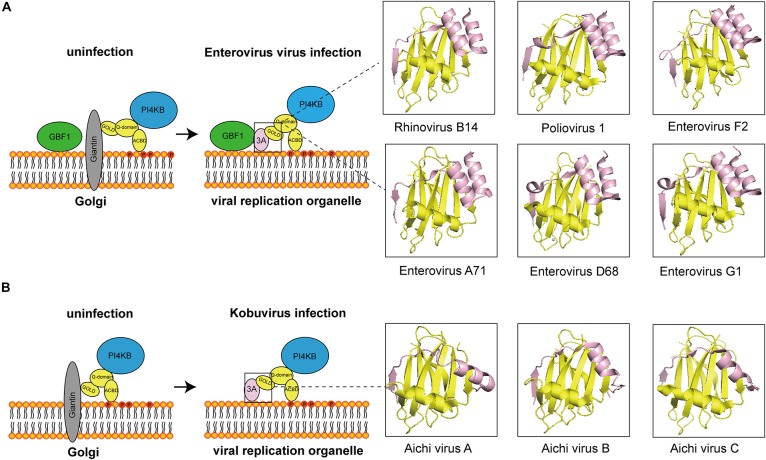
The diagram of how 3A proteins from enterovirus and kobuvirus hijack ACBD3. **(A)** In uninfected cells, the GOLD domain of ACBD3 binds to giantin that is anchored to the Golgi membrane. GBF1 is localized in membrane. PI4KB is localized to the Golgi through interaction with the Q-domain of ACBD3. In enterovirus-infected cells, viral proteins 3A compete with giantin for binding to ACBD3. 3A also interacts with GBF1. The 3C/ACBD3/PI4KB complex is formed in viral ROs. The crystal structures of ACBD3 GOLD domain in complex with 3A protein from human Rhinovirus B14 (Protein Data Bank ID: 6HLT), human Poliovirus 1 (Protein Data Bank ID: 6HLV), bovine human Enterovirus F2 (Protein Data Bank ID: 6Q68), human Enterovirus A71 (Protein Data Bank ID: 6HLW), human Enterovirus D68 (Protein Data Bank ID: 6HLN), and porcine Enterovirus G1 (Protein Data Bank ID: 6Q69) were shown in the right. **(B)** In uninfected cells, the GOLD domain of ACBD3 binds to giantin that is anchored to the Golgi membrane. PI4KB is localized to the Golgi through interaction with the Q-domain of ACBD3. In Kobuvirus-infected cells, viral proteins 3A compete with giantin for binding to ACBD3. The 3C/ACBD3/PI4KB complex is formed in viral ROs. The crystal structures of ACBD3 GOLD domain in complex with 3A protein from human Aichi virus A (Protein Data Bank ID: 5LZ3), human Aichi virus B (Protein Data Bank ID: 5LZ6), porcine Aichi virus C (Protein Data Bank ID: 6Q67) were shown in the right.

At present, most of the viruses that interact with ACBD3 are from the *Picornaviridae* family and are PI4P-dependent viruses. In most of viruses, ACBD3 plays a central role in recruiting 3A and PI4KB to produce PI4P. The structures of how 3A proteins from enterovirus and kobuvirus hijack ACBD3 have been elucidated, which is illustrated in Figure 1. How does viral-induced PI4P facilitate viral replication? The original hypothesis is that PI4P recruits the viral RNA-dependent RNA polymerase (RdRP) 3D^pol^ (Hsu et al., 2010). However, a recent study indicated that negative charge and the membrane-tethered 3B protein worked together to recruit 3D^pol^ (Dubankova et al., 2017). Interestingly, researchers found that PI4P recruited OSBP to accumulate cholesterol on viral ROs (Arita, 2014; Roulin et al., 2014). Recent studies on PI4KB-resistant enteroviruses suggest that cleavage of viral 3AB protein and development of viral ROs are the targets of PI4KB/OSBP pathway in enterovirus replication (Arita, 2016; Melia et al., 2017; Arita and Bigay, 2019). In addition to 3D^pol^-recruitment models, these observations provide a fair view on the understanding of PI4KB/OSBP pathway. In summary, ACBD3 could be a scaffold responsible for viral ROs formation, representing a new direction for future research.

The role of ACBD3 in the replication of other viruses such as the African swine virus is less well-described. ACBD3 has been identified as a binding partner for other viruses. Yeast two-hybrid screening revealed an interaction between ACBD3 and NS3 from duck Tembusu virus (DTMUV) (Wang et al., 2019). ACBD3 associates with the Hantavirus non-structural protein (Ronnberg et al., 2012). Further research is needed to explore whether ACBD3 plays a role in additional viruses.

## Author Contributions

LZ conceived the work. YL, SS, and LZ wrote the manuscript and approved the final version for publication.

## Conflict of Interest

The authors declare that the research was conducted in the absence of any commercial or financial relationships that could be construed as a potential conflict of interest.
